# CD8 apoptosis may be a predictor of T cell number normalization after immune reconstitution in HIV

**DOI:** 10.1186/1479-5876-5-9

**Published:** 2007-01-30

**Authors:** Dorothy E Lewis, Kimber L Gross, Martine M Diez, Maria L Martinez, Helen N Lukefahr, Claudia A Kozinetz, Roberto C Arduino

**Affiliations:** 1 Department of Immunology, Baylor College of Medicine, Houston, Texas, USA; 2 Department of Mathematics, University of Houston, Houston, Texas, USA; 3 Department of Medicine, University of Texas-Houston Health Sciences, Houston, Texas, USA; 4 Department of Pediatrics, Baylor College of Medicine, Houston, Texas, USA; 5 Cambridge University, UK; 6 Baylor College of Medicine/University of Texas Center for AIDS Research, Houston, Texas, USA

## Abstract

**Background:**

As part of the Houston Vanguard study, a subset of 10 patients randomized to receive IL-2 therapy were compared to 4 patients randomized to not receive IL-2, for markers of T cell activation and death during the first three cycles of IL-2. All patients were treated with combination antiretroviral therapy (ART) and were virally suppressed. The purpose of the study was to examine the role of CD8+ T cell death in responses to ART and IL-2 therapy.

**Methods:**

Lymphocytes were examined at Day 0, 5 and 30 days during three cycles of IL-2 therapy. CD25, CD38, HLA-DR expression and annexin (cell death) were examined on CD4 and CD8 subpopulations. Follow up studies examined CD4 levels and CD4:CD8 reconstitution after 6 years using both univariant and multivariate analyses.

**Results:**

Human lymphocytes responded to IL-2 therapy by upregulation of CD25 on CD4^+ ^T cells, leading to an increase in CD4 cell counts. CD8^+ ^T cells did not increase CD25 expression, but upregulated activation antigens (CD38 and DR) and had increased death. At baseline, 7 of the 14 patients had high CD8^+ ^T cell apoptosis (mean 17.0% ± 6.0). We did an exploratory analysis of immune status after six years, and found that baseline CD8^+ ^T cell apoptosis was correlated with CD4 cell count gain beginning two years post enrollment. Patients with low levels of CD8^+ ^T cell apoptosis at baseline (mean 2.2% ± 2.1) had significantly higher CD4 cell counts and more normalized CD4:CD8 ratios than patients with high CD8^+ ^T cell apoptosis (mean CD4 cell counts 1,209 ± 164 vs 754 ± 320 cells/mm^3^; CD4:CD8 ratios 1.55 vs. 0.70, respectively).

**Conclusion:**

We postulate that CD8^+ ^T cell apoptosis may reflect inherent activation status, which continues in some patients even though viral replication is suppressed which influences the ability of CD4^+ ^T cells to rebound. Levels of CD8^+ ^T cell apoptosis may therefore be an independent predictor of immune status, which should be shown in a prospective study.

## Background

IL-2 therapy leads to dose-dependent, sustained CD4 cell count increases in most HIV-infected patients treated with antiretroviral therapy (ART) [1,2]. Early increases in both CD4^+ ^and CD8^+ ^T cell subsets occurs, but only the CD4^+ ^T cell population maintains higher numbers over the long-term [3,4]. The mechanism may involve the ability of CD4^+ ^T cells to better upregulate the high affinity IL-2 receptor, CD25 or it may involve preferential survival of CD4^+ ^T cells. Recent data suggests that the expansion of CD25 expressing cells after IL-2 administration is associated with less proliferation of T cells in the long-term (12 months) as measured by both Ki67 expression [3,4] and after in vivo labeling of DNA [4]. It is suggested that IL-2 actually allows CD4 expansion to be maintained by decreasing immune activation levels. One possible explanation is that IL-2 causes the expansion of regulatory CD4^+ ^CD25^+ ^T cells, which might limit subsequent CD4 T cell activation [5].

Other predictors of the amount of CD4 restoration after IL-2 include the CD4 nadir and the age of the patient [6,7]. The lower the CD4 nadir, the fewer numbers of CD4^+ ^T cells return after IL-2 therapy. Likewise, the older the IL-2 treated patient, the less likely they are to respond well to IL-2, especially after three cycles [7]. Non-responders to IL-2 therapy also tend to have more T cell proliferation at baseline, as well as reduced numbers of recent thymic emigrants, suggesting increased turnover of the peripheral T cell pool [6,7].

In another study, a predictor of the amount of CD4^+ ^T cell return after IL-2 therapy was being non-white; this group of individuals had almost a 200 cells/mm^3 ^larger increase in CD4 cell counts than the white group studied from a cohort of more than 250 patients [6].

Another feature of HIV pathogenesis that might influence T cell normalization after immune reconstitution is the increased cell death of both CD4^+ ^and CD8^+ ^T cells that is commonly seen in HIV-infected patients. Increased CD4^+ ^T cell death could be due to HIV infection directly or bystander CD4^+ ^T cells may die via multiple mechanisms (8–10). CD8^+ ^T cell death is likely due to increased activation [11-13]. In terms of reduction of lymphocytic apoptosis after ART, several studies show a reduction; however, apoptosis levels do not return to normal levels, but remain elevated in most [14-16].

One study showed that the levels of T cell apoptosis, especially CD8^+ ^T cell apoptosis, was associated with recovery of CD4^+ ^T cell numbers after ART, so that higher levels of CD8^+ ^T cell apoptosis was associated with fewer CD4^+ ^T cells after therapy [17]. This is consistent with our own cross-sectional observation that patients with fewer CD4^+ ^T cells also had more CD8^+ ^T cell apoptosis, whether or not they were on ART therapy [18]. Another study has shown that the amount of CD4^+ ^T cell apoptosis was reduced after four weeks with or without IL-2 therapy, but reduction of CD8^+ ^T cell apoptosis levels took 24 weeks to develop [19]. However, levels of lymphocytic apoptosis were still higher than in controls and was similarly elevated in both ART treated and ART plus IL-2 treated patients [19,20]. When lymph nodes were examined for levels of T cell death before and after ART, low levels of CD8^+ ^T cell apoptosis remained after ART, which were reflective of continuing low levels of viral replication [21].

The present Houston Vanguard sub-study examines a small group of subcutaneously IL-2 (scIL-2) treated patients. Patients were randomized from October 1998 to May 1999. An exploratory analysis of immunologic status was assessed an average of six years after randomization. We found that, in this small group of patients, the level of spontaneous CD8^+ ^T cell apoptosis at baseline before commencing scIL-2 therapy was associated with both CD4 cell counts as well as CD4:CD8 ratio normalization after six years. These differences in T cell numbers based on initial CD8^+ ^T cell apoptosis levels appeared after two years of scIL-2 therapy and were maintained until the current analysis.

## Methods

### Study population

To participate in the Houston Vanguard study, subjects had to be at least 18 years old, have documented HIV-1 infection and a CD4 cell count ≥ 350 cells/mm^3 ^at screening. The Karnofsky performance status had to be ≥ 80 and the patients had to be receiving antiretroviral therapy for at least 7 days prior to starting scIL-2 therapy. Women of childbearing potential were required to have a negative pregnancy test before initiation of scIL-2 therapy. At baseline, all but one of the individuals had plasma HIV RNA levels below 500 copies/mL. That single individual had a viral load of 2,007 copies/mL. The main exclusion criteria for this study included any concurrent or prior history of AIDS-defining illnesses, malignancy requiring systemic therapy within the prior 5 years, concomitant use of systemic corticosteroids, chemotherapy or experimental cytotoxic drugs, or current or prior history of autoimmune and/or inflammatory diseases and breastfeeding. This study was approved by the institutional review board (IRB) at The University of Texas Health Science Center at Houston and the National Institute of Allergy and Infectious Disease (NIAID) IRB. All subjects provided written informed consent.

### Trial design

The Houston Vanguard study was an open-label, randomized trial designed with a target sample size of 72 subjects with CD4 cell count change from baseline as the primary endpoint. Subjects were sequentially randomized to three groups of 24 (12 in each treatment group, scIL-2 or antiretroviral alone). The first group of 24 subjects received 1.5 Million International units (MIU) per dose of scIL-2 plus antiretroviral therapy or antiretroviral therapy alone (control); the second group of 24 subjects was randomized to 4.5 MIU scIL-2 or control; and the third group was randomized to 7.5 MIU of scIL-2 or control. For the purpose of this immunology sub-study of the Houston Vanguard, consecutive patients who volunteered to participate in the sub-study were enrolled: five patients from the 1.5 MIU per dose cohort (low dose), five patients from the 7.5 MIU per dose cohort (high dose) and four from the no IL-2 cohort (control group).

### scIL-2 treatment

Subcutaneous IL-2 (aldesleukin [Proleukin]; Chiron, Emeryville, CA) was administered for 5 days every 8 weeks for a minimum of three cycles in addition to continued antiretroviral therapy. Dosing with scIL-2 was begun at 1.5 MIU. This dose was escalated to 4.5 MIU and then to 7.5 MIU when at least 9 of the 12 subjects had completed all doses of the first cycle with scIL-2 at a lower dose level in the absence of dose-limiting toxicities. Dose reductions of scIL-2 were allowed for dose-limiting toxicities defined as grade IV toxicities (as defined in the National Institutes of Health, Division of AIDS Toxicity Table for Grading Adverse Events), investigator's assessment or subject tolerance.

After completion of three cycles, subjects were enrolled in an extension phase (months 6 to 12) and, thereafter, rolled-over into the ESPRIT study (Evaluation of subcutaneous Proleukin^® ^in a Randomized International Trial). Additional cycles of scIL-2 were encouraged to maintain CD4 cell counts more than twice baseline or greater than 1,000 cells/mm^3^. For subjects randomized to 1.5 and 4.5 MIU, dose escalation was allowed by increments of 1.5 MIU per dose up to a maximum of 7.5 MIU. Subjects receiving less than 4.5 MIU were permitted to dose escalate by increments of 1.5 to 3.0 MIU upon the investigator's discretion. Dosage could be reduced by 1.5 MIU decrements (to a minimum dose of 1.5 MIU) if the assigned dose was not tolerated. During the extension phase and roll-over ESPRIT no control subjects were treated with scIL-2 at any dose. The cumulative amount of IL-2 given to the 10 IL-2 treated patients in this study ranged between 165–570 MIU, with a mean of 302 ± 132.

### Follow-up studies

Of the 14 originally enrolled in the study, 11 were still followed by Year 6. One patient with low CD8^+ ^T cell apoptosis at baseline dropped out before Year 2, and two of the non-IL-2 patients withdrew consent, one before Year 1 and one before Year 3. Both of these patients had high levels of CD8^+ ^T cell apoptosis at baseline.

### Measurements of viral load/Lymphocyte aubsets

Plasma HIV RNA levels were quantified by branched DNA signal amplification assay (Quantiplex; Chiron Emeryville, CA, Version 2.0, lower limit of detection 500 copies/mL; and 3.0, lower limit of sensitivity 50 copies/mL). CD4 and CD8 counts were done by commercial flow cytometry laboratories using CAP defined criteria.

### Measurements of activation and death on lymphocyte subpopulations

Peripheral blood was separated using Ficoll gradients. The cells were washed in PBS, counted and then stained for cell surface markers and Annexin V to determine levels of apoptotic cells. CD4, CD8, CD25, CD38, and DR antibodies were purchased from BD Pharmingen (San Jose, CA) and Annexin V was obtained from Biosource International (Camarillo, CA). The cells were analyzed using a Beckman Coulter XL2 flow cytometer. Twenty-thousand events were collected, gated on "viable" light scatter, and fluorescence distributions collected and analyzed and expressed as percentages or mean fluorescence intensity [18].

### Statistical analysis

Group means and standard deviations of study variables: CD4, CD8, and CD4:CD8 ratios were obtained for both low and high annexin groups. Tests for normality were performed. Normally distributed data were analyzed using the t-test and one way repeated measures analysis of variance. A difference of p < 0.05 was considered significant. For missing data, the last value carried forward (LVCF) principle was applied to the analyses. A multivariate analysis of the data was also performed using generalizing estimating equations (GEE) a technique appropriate for longitudinal data with a small number of subjects [22]. Variables entered, separately at first, in the models as covariates of annexin included age at randomization, age at diagnosis and race. CD4 nadir was not included because it is a component of the outcome variables (CD4 T cell count and CD4: CD8 ratio). Age was included as a continuous variable. Race was dichotomized to black and non-black.

## Results

### Initial studies

Initial studies conducted after randomization examined five scIL-2 treated patients at high dose (7.5 MIU per dose) and five on low dose (1.5 MIU per dose) for three cycles of scIL-2 over a six month period. These groups were compared to four patients treated with ART who did not receive scIL-2 therapy (controls). Results show that the absolute CD4 cell counts increased in all three groups and that the ending CD4:CD8 ratios were significantly different between groups (Table 1). Those receiving the highest dose of scIL-2 had the biggest increase in the CD4:CD8 ratio.

**Table 1:** Increase in CD4 Cell Counts and CD4:CD8 Ratio After 3 scIL-3 Cycles.

**scIL-2 Dosage**	**Change in CD4 Number**	Increase in Ratio*
7.5 MIU(n = 4)	863 ± 434	0.88 ± 0.15
1.5 MIU(n = 5)	554 ± 458	0.53 ± 0.09
Control (n = 3)	442 ± 284	0.23 ± 0.14

* Day 65 in treated and week 24 in controls.

All different from each other (p = < 0.01). All patients increased their CD4:CD8 ratio, with the high dose IL-2 patients responding best. There is one missing endpoint in the high dosage at Day 65 and one in the group that received no IL-2 at 24 weeks.

**Figure 1:**
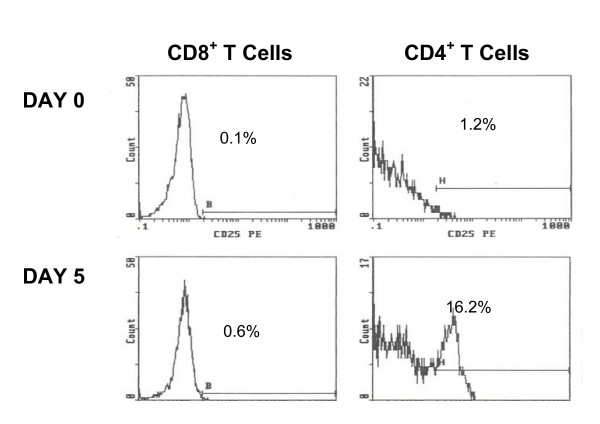
**Increase in CD25 Expression on CD4^+ ^T Cells 5 Days after IL-2 Therapy Initiation**. Histogram of a representative response of CD4^+ ^and CD8^+ ^T cells to scIL-2. Shown are staining of CD4^+ ^and CD8^+ ^T cells with CD25 at Day 0 and after 5 days of self administered scIL-2. Only the CD4^+^T cells show an increase of CD25 after 5 days.

**Figure 2:**
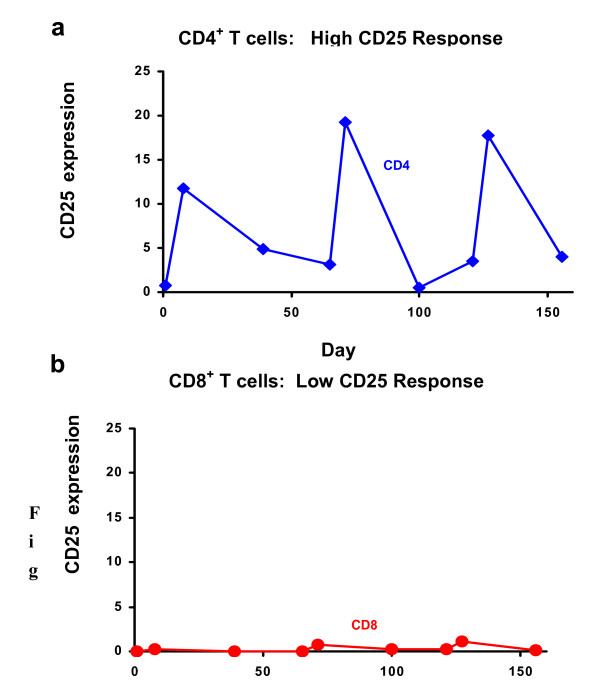
**CD4^+ ^T Cells Respond to IL-2 by Up-regulating CD25 Expression 5 Days after Every Cycle**. **a and b**. IL-2 induces more CD25 expression on CD4^+ ^T cells. Values are percentage of (a) CD4^+ ^or (b) CD8^+ ^T cells which express CD25. Representative low-dose recipient shown. Patient received scIL-2 on days 1–5, 65–69 and 121–125

**Figure 3:**
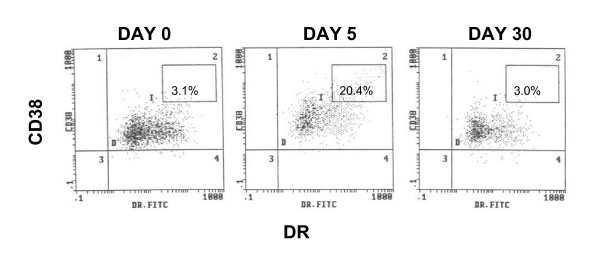
**Increase in CD38+DR+ CD8^+ ^T Cells 5 Days after scIL-2 Therapy Initiation**. Cytograms of CD38 and DR expression on CD8^+ ^T cells during a single cycle of scIL-2. Both the percentage of CD38DR^bright ^cells increases as well as the MFI of CD38 on the whole population. (CD38 MFI DAY 0 = 7.41, DAY 5 = 20.9, DAY 30 = 7.46)

Similar to what others have observed, there was an increase of CD25, the high affinity IL-2 receptor, on the CD4^+ ^T cells five days after scIL-2 therapy, as shown in Figure 1. The percentages and numbers of CD25^+ ^CD4^+ ^T cells before and after three cycles of scIL-2 therapy in the three groups are shown in Tables 2 and 3. Such an increase in CD25 expressing cells was not observed for CD8^+ ^T cells (Figure 2). Rather, at Day 5 there was a sharp increase in CD8^+ ^CD38^+ ^DR^+ ^bright cells, as shown in Figure 3. The high levels of these cells returned to baseline by Day 30 of each cycle.

**Table 2:** Increases in CD4^+ ^CD25^+ ^T Cells after IL-2 Therapy; Percentages of CD25^+ ^CD4^+ ^T Cells

**scIL-2 dose**	**Baseline**	**After 3 Cycles**	**Change**
7.5 MIU	1.0 ± 0.7	10.7 ± 6.7	9.7 ± 6.7
1.5 MIU	0.9 ± 0.6	3.9 ± 2.5	2.9 ± 2.6
Control	1.5 ± 0.9	2.9 ± 1.4	1.5 ± 1.1

Percentages of CD25^+ ^CD4^+ ^T cells at baseline and after 3 cycles of scIL-2 therapy (week 24). The high dose IL-2 produced the greatest increase in CD25 percentages after scIL-2 administration. The high group had 4, the low group has 5 and none had 3.

**Table 3:** Increases in CD4+ T Cells after IL-2 Therapy; Numbers of CD25^+ ^CD4^+ ^T Cells

**scIL-2 dose**	**Baseline**	**After 3 Cycles**	**Change**
7.5 MIU	16.6 ± 13.0	315.4 ± 196.7	299 ± 199
1.5 MIU	12.8 ± 13.6	110.6 ± 103.9	98 ± 111
Control	22.3 ± 15.6	52.3 ± 15.3	30 ± 23

Numbers of CD25^+ ^CD4^+ ^T cells at baseline and after 3 cycles of scIL-2 therapy (week 24). The high dose IL-2 produced the greatest increase in CD25 numbers after scIL-2 administration. The high group had 4, the low group has 5 and none had 3.

**Figure 4:**
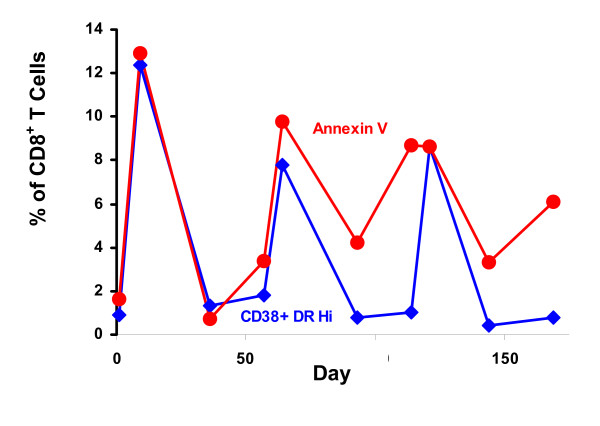
**Activation and death of CD8 T cells in a patient receiving three cycles of high dose scIL-2**. Dying CD8^+ ^T cells are activated. Values are percentage of CD8^+ ^T cells that are CD38^+ ^DR^Hi ^(diamonds) or that bind Annexin V (circles). Patient received 7.5 MIU scIL-2 on days 1–5, 57–61 and 114–118.

Next, we examined whether cells were dying at increased levels both before and after IL-2 therapy by Annexin V staining. At baseline, 7 patients had high levels of CD8^+ ^T cell apoptosis (17.0% ± 6.0) and 7 patients had low levels CD8^+ ^T cell apoptosis (2.2% ± 2.1). Four and 6 patients from each group, respectively, received scIL-2. After five days of scIL-2 therapy there was an increase in CD8 apoptosis which mirrored the amount of the CD38^+ ^DR^+ ^increase, as shown in a representative patient in Figure 4. Very little increase in CD4^+ ^T cell death was seen. We also observed increases in CD38 and DR expression on the CD4^+ ^T cells at 5 days (data not shown). The levels of CD38 and DR returned to normal on both CD4^+ ^and CD8^+ ^T cells after each cycle. There was variability in the amounts of cell death and CD38 and DR increases over the cycles, depending on the person and cycle number with no discernible pattern. In sum, our initial data is characteristic of the data in a number of published papers with larger groups of patients, except that we saw little CD4^+ ^T cell death [7,23].

### Follow-up studies

Six years after our initial study, we examined the patients for their immune status using an exploratory analysis. We compared their CD4 and CD8 cell counts and the CD4:CD8 ratios to their baseline parameters. At baseline, only 3 of the 14 patients had CD4:CD8 ratios over 1; five patients had ratios below 0.5. Examination after six years showed that 9 of 12 patients had CD4:CD8 ratios over 1 and only 2 patients had ratios below 0.5.

We next examined possible factors associated with good responses and poor responses to ART in this 6-year period, independently of scIL-2 administration. We found a correlation between the percentage of Annexin V^+ ^CD8^+ ^T cells at baseline and the final CD4 cell count and CD4:CD8 ratio, which indicates the extent of T cell normalization. Patients with high levels of CD8^+ ^T cell apoptosis at baseline (mean 17.0% ± 6.0) did not normalize their CD4 cell counts or CD4:CD8 ratios after six years of follow-up; whereas, patients with low levels of CD8^+ ^T cell apoptosis (mean 2.2% ± 2.1) normalized their CD4 cell counts and CD4:CD8 ratio. The mean CD4 cell counts were 1,209 ± 164 vs 754 ± 320 cells/mm^3 ^and the CD4:CD8 ratios 1.55 vs. 0.70, for the low and high baseline CD8^+ ^T cell apoptosis level groups, respectively (Tables 4 and 5).

**Table 4:** CD8 Annexin V Levels at Baseline and 6 Years.

**% Annexin V levels (mean ± SD)**	**Baseline (mean ± SD)**			**6 Years (mean ± SD)**		
	
	**CD4 (cells/mm^3^)**	**CD8 (cells/mm^3^)**	**CD4:CD8 Ratio**	**CD4 (cells/mm^3^)**	**CD8 (cells/mm^3^)**	**CD4:CD8 Ratio**
**Low 2.2 ± 2.1**	625 ± 221	967 ± 386	0.76 ± 0.4	1209 ± 164	915 ± 308	1.55 ± 0.9
**High 17.0 ± 6.0**	527 ± 132	1234 ± 660	0.56 ± 0.36	754 ± 320	1172 ± 456	0.76 ± 0.4
**p-value**	p = 0.331	p = 0.374	p = 0.327	p = 0.001	p = 0.240	p = 0.038

CD4 values, CD8 values, and CD4:CD8 ratios were recorded at baseline and 6 years for the low and high apoptosis groups. At baseline, a parametric t test showed no significance between low and high CD8 apoptosis levels for CD4, CD8 or CD4:CD8 ratios. However, after 6 years, a significant difference was observed between the high and low annexin groups with respect to CD4 values and CD4:CD8 ratios with p values of ≥ 0.001 and ≥ 0.037. CD8 was not significantly different at any time point.

**Table 5:** CD8 Annexin V Levels at Baseline and 6 Years.

**% Annexin V levels (mean ± SD)**	**CD4 (cells/mm^3^)**			**CD8(cells/mm^3^)**			**CD4:CD8 Ratio**		
	
	**Baseline**	**6 YR**	**P-value**	**Baseline**	**6 YR**	**P-value**	**Baseline**	**6 YR**	**P-value**
**Low 2.2 ± 2.1**	625 ± 221	1209 ± 164	0.001	967 ± 386	915 ± 308	0.766	0.76 ± 0.4	1.55 ± 0.9	0.038
**High 17.0 ± 6.0**	527 ± 132	754 ± 320	0.098	1234 ± 660	1172 ± 456	0.728	0.56 ± 0.36	0.7 ± 0.34	0.215

A repeated measures ANOVA showed a significant difference in the low annexin group for CD4 values from baseline to year 6 with a p value of p ≥ 0.001. The high annexin group CD4 values were not significant from baseline to year 6 with a p value of ≥ 0.098. CD8 values in both the low and high apoptosis groups were not significant for the baseline or 6 year analysis. The CD4:CD8 ratio for the low apoptosis group was significant with a p value of ≥ 0.038, but the ratio for the high apoptosis group was not significant (p ≥ 0.215) after 6 years.

**Figure 5:**
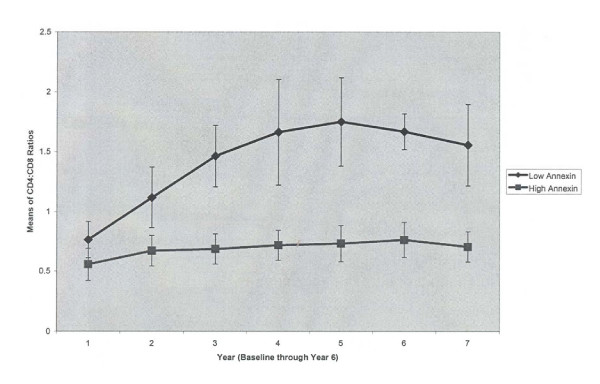
**CD4:CD8 Ratios over Time in Low versus High CD8 Apoptosis Groups**. The low baseline CD8^+ ^T cell annexin group has a higher mean CD4:CD8 ratio after six years than high baseline CD8^+ ^T cell annexin group. The results are the mean values of CD4:CD8 ratio ± SD. A repeated measures ANOVA indicated a statistically significant difference (p = 0.041).

To determine when during the follow-up period this effect occurred, we examined CD4:CD8 ratios in the high and low CD8^+ ^T cell apoptosis groups over time and found the results shown in Figure 5. By two years of follow-up, the high and low CD8^+ ^T cell Annexin V positive groups were separated by differences in their CD4:CD8 ratios, which were differentially maintained to the present.

We also examined this small group of patients for predictors of CD4 cell count increase and CD4:CD8 ratio normalization. A summary is shown in Table 6. There were no differences in age, sex, duration of HIV infection or co-infections with HBV or HCV. There was a trend of lower CD4 nadir among patients in the high vs. low CD8 apoptosis groups (x¯
 MathType@MTEF@5@5@+=feaafiart1ev1aaatCvAUfKttLearuWrP9MDH5MBPbIqV92AaeXatLxBI9gBaebbnrfifHhDYfgasaacH8akY=wiFfYdH8Gipec8Eeeu0xXdbba9frFj0=OqFfea0dXdd9vqai=hGuQ8kuc9pgc9s8qqaq=dirpe0xb9q8qiLsFr0=vr0=vr0dc8meaabaqaciaacaGaaeqabaqabeGadaaakeaacuWG4baEgaqeaaaa@2E3D@ = 299 ± 144 vs. 178 ± 93, respectively; median was 384 versus 203), but this difference was not statistically significant.

**Table 6:** Baseline Characteristics of HIV Patients in Study.

Annexin levels at baseline	Low	High	Total
N	7	7	14
Age at randomization (years; mean)	35	37	36
Age at HIV diagnosis (year; mean)	33	33	33
Female (n)	2	2	4
Race			
Black (n)	4	0	4
White (n)	1	2	3
Hispanic (n)	2	5	7
Likely mode of infection*:			
Sexual contact, same sex (n)	3	4	7
Sexual contact, opposite sex (n)	4	3	7
Injection drug use(n)	1	3	4
CD4+ (cells/mm3; median)	619	590	605
CD4+ nadir (cells/mm3; median)	384	203	219
HIV RNA <500 copies/mL (n)	6	7	13
Time since first prescribed ART (months; median)	14	20	17
Hepatitis B co-infection (n)	2	4	6
Hepatitis B co-infection (n)	1**	1	2
Hepatitis serology unknown (n)	2	2	4

* Subjects reported more than one mode of infection.

** Subject co-infected with HBV and HCV.

Examination of the ethnic distribution of the two groups revealed that there were one white, two Hispanics and four Blacks in the low apoptosis group and two whites and 5 Hispanics in the high apoptosis group. Thus, in this small sample, all four Blacks recovered CD4 numbers better after six years. Similar results were suggested in a previous much larger study, which examined non-whites, most of which were Black (70%) [6].

We next used GEE modeling to exam the outcomes over time. Baseline annexin had a significant inverse relationship with CD4 T cell count and ratio over time (p ≥ 0.02). When age at randomization was entered into the model, baseline annexin remained a significant predictor of both CD4 T cell count over time (p ≥ 0.04) and CD4:CD8 ratio (p ≥ .0.05). A similar finding occurred for age at HIV diagnosis, so that baseline annexin remained a significant predictor of CD4+ cell count over time (p ≥ 0.03) and CD4:CD8 ratio (p ≥ .0.04). When race was entered into the model, annexin was no longer independently related to CD4 T cell and CD4:CD8 ratio over time. When annexin, age and race were entered into the model, age at diagnosis was significant predictor of CD4 T cell number over time (p ≥ 0.03). Thus, this data is similar to previously reported trends with age as a predictor.

## Discussion

These results, in a small group of scIL-2 treated, virally suppressed HIV-infected patients suggest that the amount of CD8 cellular death at baseline may correlate with the ability to restore CD4 cell counts and CD4:CD8 ratios after immune reconstitution involving ART with or without additional scIL-2 therapy. These differences were detectable after two years of study enrollment. Increased CD8^+ ^T cell apoptosis was a characteristic of about 70% of previously studied patients where we found a negative correlation between high CD8^+ ^T cell apoptosis and low CD4^+ ^T cell percentages [18]. Others have also suggested that the activation level of CD8^+ ^T cells in HIV-infected patients is an independent predictor of immune status, separable from viral load and CD4 cell count [24,25]. Our data in virally suppressed patients suggest that levels of CD8^+ ^T cell apoptosis also may independently predict response to therapies, including scIL-2, by not only increasing the absolute number CD4 cells, but perhaps by regulating the levels of CD8^+ ^T cell expansion.

The role of lymphocytic apoptosis in the response to ART with or without IL-2 has been studied by several groups. Most suggest apoptosis levels of both CD4^+ ^and CD8^+ ^T cells tend to be reduced after ART (14–16). However, in multiple studies, the levels of apoptosis still remains higher than in controls, which likely indicates the presence of continued immune activation which was previously demonstrated in the lymph nodes [21]. IL-2 therapy seems to have little effect on apoptosis levels in the long-term. Preferential apoptosis of activated CD8^+ ^T cells was also seen by another group at 5 days post scIL-2 therapy similar to our observations [23]. However, there was little difference in spontaneous CD8^+ ^T cell apoptosis levels with time, suggesting that IL-2 did not change the inherent level of CD8^+ ^T cell apoptosis.

The work that most closely approximates the work reported here followed 17 patients during the first 2–3 years of ART and showed that lymphocyte apoptosis was reduced most in those who had the best response to therapy [26]. Although the beginning levels of apoptosis appear different between good responders and poor responders in that study, the authors did not note that this might be an important variable for the outcome. They do show a negative correlation between the percentage of Annexin V^+ ^CD8^+ ^T Cells and CD4^+ ^T cells over two years of ART therapy. Another difference in our work is that the patients were already virally suppressed when baseline apoptosis was measured. Our data, with a small group of patients indicates that the starting level of apoptosis reflects long term outcome to therapy and that the level of CD8 T cell death is independent of viral load.

IL-2 is known as the consummate T cell growth factor; however, IL-2 not only enhances proliferation and survival of T cells, it can also signal death in susceptible cell populations. We suggest that the activated CD38^+ ^DR^+ ^population, which dies after acute administration of IL-2, is indicative of this type of response to IL-2 in vivo. Since CD8^+ ^T cells do not upregulate CD25, it is likely they respond to IL-2 via binding to CD122, (B chain IL-2R), which at the time of these studies was not commercially available. Of importance is that we saw no increase in CD4^+^T cell death, even though there was CD38^+ ^DR^+ ^activation marker upregulation in the treated people at Day 5 of the scIL-2 cycle. This is in contrast to the conclusions of another report; however, the data in that manuscript also clearly show more apoptosis in the activated CD8^+ ^T cells than the CD4^+ ^T cells after scIL-2 administration at Day 5 [23]. We think that activation of T cells by IL-2 likely leads to differential outcomes in CD4^+ ^vs. CD8^+ ^T cell subpopulations. Recent data, which examined DNA turnover in CD4^+ ^and CD8^+ ^T cell subsets of HIV-infected people after long-term scIL-2 therapy, are supportive of this idea because IL-2 therapy enhanced CD4^+ ^T cell survival, but not CD8^+ ^T cell survival [4].

The mechanism responsible for enhanced normalization of CD4 cell counts in those with less CD8^+ ^T cell death is unclear. It may be associated with a better overall CD4 cell count at the beginning point in the low CD8^+ ^T cell apoptosis group. The CD4 cell count nadir in this group was about 100 cells/mm^3 ^higher and the median was higher than in the high CD8^+ ^T cell apoptosis group, which is consistent with our previous observations that a high level of CD8^+ ^T cell apoptosis was correlated with fewer CD4^+ ^T cells [18].

Our interpretation of this data is that the amount of CD8^+ ^T cell apoptosis at baseline indicates existing immune activation to HIV antigens, which can continue in some patients, even without detectable viremia [14-16]. CD8^+ ^T cells, therefore, get activated and die at an increased level in some individuals. Even though CD4 cell count increases in IL-2 treated patients, the amount of proliferation in the long term actually becomes reduced as shown in two studies [3,4]. It has been suggested that proliferation, as measured by Ki67 staining, found in cells in the SG_2_M phase of the cell cycle, is similar to measurements of activation using CD38 and DR expression [27]. Hence, the better long-term outcome in those with less apoptosis might be due to less inherent activation of cells from these patients [28,29]. In the one IL-2 long-term non-responder patient studied by Kovacs, et al [4], the DNA turnover of both CD4^+ ^and CD8^+ ^T cells remained high, even after 22 cycles of scIL-2, indicating that chronic activation in some people is not relieved by IL-2 therapy. Of interest is that this patient also had about a log_10 _more HIV RNA (32,873 copies/mL) than other patients. However, we saw no correlation in our small study with viral load, which remained undetectable or barely detectable throughout the study [30]. We did not have access to information on viral set point in most of the patients. Non-responders to IL-2 also have more T cell activation at baseline as shown by others [3,4], which could also be reflected by higher levels of CD8^+ ^T cell death. The underlying reason for this is not clear, but could be related to inherent host differences or a "set point" of CD4 cell count, below which an enhanced CD8^+ ^T cell apoptosis is triggered.

Another possible mechanism to account for the role of CD8^+ ^T cell apoptosis relates to what drives the proliferation of T cells. That is, homeostatic mechanisms, like that driven by IL-7, are one way that T cell proliferation is driven. By contrast, activation-induced proliferation is another mechanism whereby T cells divide. We think it likely that the predominant form of CD8 proliferation occurring in those with high CD8^+ ^T cell apoptosis is activation-induced, rather than homeostatic-driven proliferation, because death occurs mainly with activation induced proliferation, not homeostatic driven proliferation [31]. This may account for the slightly higher CD8 cell counts in those with high apoptosis both before and after IL-2 therapy. These individuals fail to normalize CD4 T cell numbers because CD8 activation and death continues in spite of attempts at CD4 cellular reconstitution.

## Conclusion

These data suggest that examination of CD8^+ ^T cell apoptosis might serve as an indirect measure of the ability to respond to immunoreconstitution. Our longitudinal univariate analyses showed that both age and annexin were significant predictors of CD4 T cell numbers over time. However, entry of race into the model, showed that annexin lost it's independent relationship to CD4+ T cell number over time. Examination of possible ethnic differences in response to immune reconstitution is therefore also warranted.

## Abbreviations

Interleukin-2 IL-2

Antiretroviral therapy ART

Million International Units MIU

Generalizing estimating equations GEE

## Competing interests

The author(s) declare that they have no competing interests.

## Authors' contributions

DEL wrote the paper, RAC conducted the clinical aspects dealing with patients, CAK performed the multivariate analysis, KLG did the initial data analysis of the flow cytometry data, other's (MD, MLM, HNL) helped with patient accrual and data management.

## References

[B1] De Paoli P (2001). Immunological effects of interleukin-2 therapy in human immunodeficiency virus-positive subjects. Clin Diagn Lab Immunol.

[B2] Marchetti G, Franzetti F, Gori A (2005). Partial immune reconstitution of highly active antiretroviral therapy: can adjuvant interleukin-2 fill the gap?. J Antimicrob Chemother.

[B3] Sereti I, Anthony KB, Martinez-Wilson H, Lempicki R, Adelsberger J, Metcalf JA, Hallahan CW, Follmann D, Davey RT, Kovacs JA, Lane C (2004). IL-2-induced CD4^+ ^T-cell expansion in HIV-infected patients is associated with long-term decreases in T-cell proliferation. Blood.

[B4] Kovacs JA, Lempicki RA, Sidorov I, Adelsberger JW, Sereti I, Sachau W, Nelly G, Metcalf JA, Davey RT, Falloon J, Polis MA, Tavel J, Stevens R, Lambert L, Hosack DA, Bosche M, Isaac HJ, Fox SD, Leitman S, Baseler MW, Masur H, Di Mascio M, Dimitrov DS, Lane HC (2005). Induction of prolonged survival of CD4^+ ^T lymphocytes by intermittent IL-2 therapy in HIV-infected patients. J Clin Invest.

[B5] Seriti I, Imamichi H, Natarajan V, Imamichi T, Ramchandani MS, Badralmaa Y, Berg SC, Metcalf JA, Hahn BK, Shen JM, Powers A, Davey RT, Kovacs JA, Shevach EM, Lane HC (2005). In vivo expansion of CD4^+ ^CD45RO^-^CD25^+ ^T cells expressing foxP3 in IL-2-treated HIV-infected patients. J Clin Invest.

[B6] Markowitz N, Bebchuk JD, Abrams DI (2003). Nadir CD4^+ ^T cell count predicts response to subcutaneous recombinant interleukin-2. Clin Infect Dis.

[B7] Marchetti G, Meroni L, Molteni C, Bandera A, Franzetti F, Massimo G, Mauro M, Mario C, Andrea G (2004). Interleukin-2 immunotherapy exerts a differential effect on CD4 and CD8 T cell dynamics. AIDS.

[B8] Alimonti JB, Ball TB, Fowke KR (2003). Mechanisms of CD4^+ ^T lymphocyte cell death in human immunodeficiency virus infection and AIDS. J Gen Virol.

[B9] Badley AD, Dockrell D, Simpson M, Schut R, Lynch DH, Leibson P, Paya CV (1997). Macrophage-dependent apoptosis of CD4^+ ^T lymphocytes from HIV-infected individuals is mediated by FasL and tumor necrosis factor. J Exp Med.

[B10] Finkel TH, Tudor-Williams G, Banda NK, Cotton MF, Curiel T, Monks C, Baba TW, Ruprech RM, Kupfer A (1995). Apoptosis occurs predominantly in bystander cells and do not in productively infected cells of HIV- and SIV-infected lymph nodes. Nat Med.

[B11] Decrion A-Z, Varin A, Estavoyer J-M, Herbein G (2004). CXCR4-mediated T cell apoptosis in human immunodeficiency virus infection. J Gen Virol.

[B12] Mueller YM, de Rosa SC, Hutton JA, Witek J, Roederer M, Altman JD, Katsikis PD (2001). Increased CD95/Fas-induced apoptosis of HIV-specific CD8^+ ^T cells. Immunity.

[B13] Tateyama M, Oyaizu N, McCloskey TW, Than S, Pahwa S (2000). CD4 T lymphocytes are primed to express Fas ligand by CD4 cross-linking and to contribute to CD8 T-cell apoptosis via Fas/FasL death signaling pathway. Blood.

[B14] de Oliveira Pinto LM, Lecoeur H, Ledru E, Rapp C, Olivier P, Gougeon ML (2002). Lack of control of T cell apoptosis under ART. Influence of therapy regimen in vivo and in vitro. AIDS.

[B15] Badley AD, Parato K, Cameron DW, Kravcik S, Phenix BN, Ashby D, Kumar A, Lynch DH, Tschopp J, Angel JB (1999). Dynamic correlation of apoptosis and immune activation during treatment of HIV infection. Cell Death Differ.

[B16] Bento JM, Lopez M, Martin JC, Lozano S, Martinez P, Gonzalez-Lahoz J, Soriano V (2002). Differences in cellular activation and apoptosis in HIV-infected patients receiving protease inhibitors or nonnucleoside reverse transcriptase inhibitors. AIDS Res Hum Retroviruses.

[B17] Hansjee N, Kaufmann GR, Strub C, Weber R, Battegay M, Erb P, Swiss HIV Cohort Study (2004). Persistent apoptosis in HIV-1-infected individuals receiving potent antiretroviral therapy is associated with poor recovery of CD4 T lymphocytes. J Acquir Immune Defic Syndr.

[B18] Lewis DE, NG Tang DS, Wang X, Kozinetz C (1999). Costimulatory pathways mediate monocyte-dependent lymphocyte apoptosis in HIV. Clin Immunol.

[B19] Pandolfi F, Pierdominici M, Marziali M, Bernardi ML, Antonelli G, Galati V, D'Offizi G, Aiuti F, IRHAN Study Group (2000). Low-dose IL-2 reduces lymphocyte apoptosis and increases naïve CD4 cells in HIV-1 patients treated with ART. Clin Immunol.

[B20] Caggiari L, Zanussi S, Bortolin MT, D'Andrea M, Nasti G, Simonelli C, Tirelli U, De Paoli P (2000). Effects of therapy with highly active anti-retroviral therapy (ART) and IL-2 on CD4+ and CD8+ lymphocyte apoptosis in HIV+ patients. Clin Exp Immunol.

[B21] Dyrhol-Riise A, Ohlsson M, Skarstein K, Nygaard S, Olofsson J, Jonsson R, Asjo B (2001). T Cell Proliferation and Apoptosis in HIV-1-Infected Lymphoid Tissue: Impact of Highly Active Antiretroviral Therapy. Clin Immunol.

[B22] Dupont WE (2002). Statistical Modeling for Biomedical Researchers: A Simple Introduction to the Analysis of Complex Data.

[B23] Seriti I, Herpin B, Metcalf J, Stevens R, Baseler MW, Hallahan CW, Kovacs JA, Davey RT, Lane CH (2001). CD4 T cell expansions are associated with increased apoptosis rates of T lymphocytes during IL-2 cycles in HIV infected patients. AIDS.

[B24] Liu Z, Cumberland WG, Hultin LE, Kaplan AH, Detels R, Giorgi J (1998). CD8+ T-lymphocyte activation in HIV-1 disease reflects an aspect of pathogenesis distinct from viral burden and immunodeficiency. J Acquir Immune Defic Syndr Hum Retrovirol.

[B25] Mildvan E, Bosch RJ, Kim RS, Spritzler J, Haas DW, Kuritzkes D, Kagan J, Nokta M, DeGruttola V, Moreno M, Landay A (2004). Immunophenotypic markers and antiretroviral therapy (IMART): T cell activation and maturation help predict treatment response. J Infect Dis.

[B26] Grelli S, d'Ettorre G, Lauria F, Montella F, Di Traglia L, Lichtner M, Vullo V, Favalli C, Vella S, Macchi B, Mastino A (2004). Inverse correlation between CD8^+ ^lymphocyte apoptosis and CD4^+ ^cell counts during potent antiretroviral therapy in HIV patients. JAC.

[B27] Paiardini M, Cervasi B, Galati D, Dominici S, Albrecht H, Sfacteria A, Magnani M, Silvestre G, Piedimonte G (2004). Early correction of cell cycle perturbations predicts the immunological response to therapy in HIV-infected patients. AIDS.

[B28] Abrams DI, Bebchuk JD, Denning ET, Davey RT, Fox L, Lane HC, Sampson J, Verheggen R, Zeh D, Markowitz NP, Beirn T (2002). Randomized, open-label study of the impact of two doses of subcutaneous recombinant interleukin-2 on viral burden in patients with HIV-1 infection and CD4^+ ^cell counts ≥ 300/mm^3^: CPCRA 059. JAIDS.

[B29] Hunt PW, Martin JN, Sinclair E, Bredt B, Hagos E, Lampiris H, Deeks SG (2003). T cell activation is associated with lower CD4+ T cell gains in human immunodeficiency virus-infected patients with sustained viral suppression during antiretroviral therapy. J Infect Dis.

[B30] Pitrak DL, Bolanos J, Hershow R, Novak RM (2001). Discordant CD4 T lymphocyte responses to antiretroviral therapy for HIV infection are associated with ex-vivo rates of apoptosis. AIDS.

[B31] Zou W, Foussat A, Capitant C, Durand-Gasselin I, Bouchet L, Galanaud P, Levy Y, Emilie D, ANRS-048 IL-2 Study Group (1999). Acute activation of CD8^+ ^T lymphocytes in interleukin-2-treated HIV-infected patients. J Acquir Immune Defic Syndr.

